# Medical News

**Published:** 1855-08

**Authors:** 


### THE MEDICAL EXAMINER.

PHILADELPHIA, AUGUST, 1855.

### MEDICAL NEWS.

The July number of the American Medical Monthly contains a very full and interesting report of the proceedings of the New York Academy of Medicine at their June meeting. The Committee on Dr. Green's paper on "Injection of the Bronchial tubes," after reading the minutes of their sessions, made their report, the conclusion of which is as follows:—

Ist. Catheterism of the air passages dates its history from the time of Hippocrates.

2d. The best evidences of the passage of an instrument into the air passages, are the rational signs.

3d. The facility of the operation depends upon the kind of instrument used; the tube having a large curve being best, and the sponge probang least adapted to enter the trachea.

4th. That there is no reliable evidence, in the opinion of your committee, that the *sponge probang* has been passed through and beyond the vocal chords.

5th. That there is no positive evidence that an instrument can be passed at will into the right or left bronchial divisions.

6th. That in the great majority of instances where injections are supposed to have been thrown into the lungs, through a tube, they have passed directly into the stomach.

7th. That as regards the utility of injections of nitrate of silver into the lungs, the facts thus far developed, in the experiments of your committee, lead them to regard the operation as one fraught with danger, as well as difficulty.

In submitting the above Report, the committee beg leave to tender their thanks to Prof. Green, and to Isaac E. Taylor, Visiting Physician of Bellevue Hospital, who kindly aided in furnishing subjects, and in making the experiments which are appended, and which form the basis of this Report.

WILLARD PARKER, M.D., *Chairman.*

JOHN O. STONE, M.D.,

ISAAC WOOD, M.D.

At an adjourned meeting of the Academy, held June 20th, Dr. Parker stated that since the last meeting of the Academy, the Report had been signed by Dr. Metcalfe, and thus had upon it the names of four of the committee (Parker, Wood, Stone, and Metcalfe.) He had received a note from Dr. Anderson signing the first part but not the second, "that is, the *cui bono.*" Dr. Stevens was present, and would answer for himself. The note of Dr. Anderson, kindly furnished to us by the writer, is as follows:

NEW YORK, *June* 14*th*, 1855.

WILLARD PARKER, M.D.:

DEAR SIR:—I still entertain the opinion I expressed at the last sitting of the committee, on the paper of Dr. Horace Green; and which I also repeated to you on the following morning; that the second division* of the matter referred to the committee had not received that investigation which would enable them intelligibly to report upon it. You will please receive this as my signature to the *first* division of the Report.

With very great respect, yours,

JAMES ANDERSON.

Dr. A. H. Stevens said that he had prepared a report, and with permission would read it.

It having been urged that the minority Report of Dr. Barker should first be read, Dr. McFarland, of Williamsburgh, stated that Dr. Stevens' was a part of the majority report, and therefore ought to be first read.

The president requested Dr. Stevens to proceed.

DR. STEVENS' REPORT.

The undersigned, one of the committee appointed to report on the subject of medicated injection into the trachea, as performed by Prof. Horace Green, not having had the opportunity to witness all of the facts in relation to this subject, that have come under the notice of his colleagues, begs to submit this his separate report. He was present at two meetings only, at Prof. Green's office, and witnessed, in addition to some very skilful applications of caustic solutions to sinous passages in the fauces, repeated introductions of the sponge probang, of the hollow tube, and of nitrate of silver injections through it.

As regards the question of the possibility of the introduction of the probang into the trachea, he did not see enough to enable him to consider the practicability of such introduction as a proved fact. But of the practicability of passing a catheter into the trachea, he was confirmed in his previous belief, that it admits of no question whatsoever. It was moreover proved that the injection of a solution of nitrate of silver in quantities equal to two fluid drachms is quite practicable with ordinary skill. In two experiments performed in his presence by Prof. Green, it was not followed by alarming symptoms, or, so far as he knows, dangerous consequences; a result quite different from what *à priori* reasoning had prepared him to anticipate. If further experience

* The utility of Injections of Nitrate of Silver into the Air Passages.

should prove its entire safety, such experience, on a very extended scale, and by numerous observers, can alone determine its therapeutic value. But those who may incline to make such experiments, will doubtless remember that a solution of two drachms of nitrate of silver, of the strength stated to have been employed, ℈jj. to ℥j. of water, would give ℈ss. of the solid caustic; enough to make an eschar through the cutis of one inch in diameter. Analogy would lead to the conclusion that so large a quantity, expending its whole force upon the tracheal, and such parts of the bronchial membrane, as it came in contact with, would be likely to induce inflammation of a grave character. The quantity of caustic actually brought into permanent contact with a stricture of the urethra is a very small fraction, probably not one-twentieth part of a grain. A stick of caustic, weighing only four or five grains, may be used daily for months, with scarcely a perceptible diminution of its bulk, even in hospital practice.

In regard to the question of the practicability of directing the solution to a particular part of the bronchial tubes, or into any cavity connected with them, at the pleasure of the operator, the reporter has seen nothing to lead him to an affirmative answer.

All which is respectfully submitted.

Alex. H. Stevens, of the Committee, &c.

We regret that our limits forbid our copying anything but the following from Dr. Barker's minority Report:

"Contenting myself, therefore, with the positive assertion that I have reliable evidence that the sponge probang has in many instances "been passed through and beyond the vocal chords," I deem it no part of my duty, as a member of *this* committee, to bring that evidence before the Academy. The committee were appointed "to enquire into and investigate the treatment proposed by Dr. Green in his Paper," read before the Academy Dec. 6th, 1854. That treatment is the injection of nitrate of silver into the bronchi, and, "under favorable circumstances," into tubercular cavities.

The following are Dr. Barker's conclusions:

In conclusion, the minority of the committee would suggest, for the acceptance of the Academy, the following propositions:

1st. Direct medication of the lungs by means of catheterism of the air tubes, was first proposed and carried into effect by our associate member, Dr. Horace Green.

2d. The operation may be performed by the dexterous surgeon with ease and facility, and with perfect safety to the patient.

3d. The results of this method of treatment, whether it has been employed in bronchial affections or in the commencement of tuberculosis, have already afforded the most gratifying indications that practical medicine will be greatly advanced by this discovery.

B. Fordyce Barker, of the committee.

---

Messrs. Lindsay & Blakiston have now in press and will shortly issue, "A Work on Epidemic Cholera, embracing its Characteristics in

General; its History and Cause; Local Preferences, and non-Contagiousness; Symptomatology, Pathology, &c." By HORATIO G. JAMESON, M. D.

---

At a special meeting of the Northern Medical Association, held July 16th, 1855, in reference to the death of Dr. M. B. Smith, the following preamble and resolutions were adopted:

*Whereas,* It has been announced to the Association that our presiding officer, Dr. Moses B Smith, "was called from works to rewards" on the morning of the 15th inst., and it appears fitting for us on this occasion to express our sentiments on account of the bereavement, and our opinion of his high worth: therefore

*Resolved* That in the decease of our venerable and lamented President, Dr. M. B. Smith, (who was one of the earliest members in our organization, and most assiduous in his attention to the welfare of the Association, in every respect,) we have sustained an irreparable loss.

*Resolved,* That we most respectfully and sincerely tender our warmest sympathy to his family in the present affliction.

*Resolved,* That as a last token of our regard to his departed worth, we will attend the funeral to-morrow afternoon in our associate capacity.

*Resolved,* That a copy of the above proceedings, signed by the Vice-President and Secretary, be sent to the family of the deceased.

*Resolved,* That a copy be also sent to the editors of the Medical Examiner and Medical News, with the request to publish.

JOHN RHEIN, Vice President.
J. HENRY SMALTZ, Sec. pro tem.

